# *Inter Arma, Judicialis:* Legal Bricolage and the Agency of Ukrainian Judiciary in Wartime

**DOI:** 10.1177/09646639251396506

**Published:** 2025-12-03

**Authors:** Agnieszka Kubal, Hanna Oliinyk

**Affiliations:** Centre for Socio-Legal Studies, University of Oxford, UK

**Keywords:** Ukraine, judiciary, war, international human rights law, Namibia exception, transitional justice

## Abstract

This paper investigates how Ukrainian judges respond to the challenges of wartime adjudication following Russia's invasion. Based on 17 interviews with judges across Ukraine, it explores their adaptive legal strategies amid uncertainty and institutional strain. The study conceptualises judges as legal bricoleurs, who drawing on international human rights norms – such as the Namibia exception and the European Court of Human Rights caselaw – balance civil liberties with national security. It argues that war has served not only as a site of crisis but also of legal creativity, fostering engagement with international law and laying groundwork for Ukraine's post-war transitional justice and alignment with global legal standards.

## Introduction

‘Inter arma silent leges’ – in times of war, the law falls silent – is a Latin maxim attributed to Cicero, signifying that in times of war or armed conflict, the ordinary rules and regulations of peace are temporarily set aside in favour of the needs and necessities of conflict (Fellmeth and Horwitz, 2011). The reality of today's armed conflicts is that they are already governed by agreed ‘rules of conduct’. International humanitarian law (IHL), beginning with the Geneva Conventions of 1864 and further expanded through the Hague Conventions (1899 and 1907), the 1949 Geneva Conventions, and the 1977 Additional Protocols, has developed significantly over the past century (Darcy, 2014). Jurisprudence from international tribunals set up in the aftermath of conflicts – most notably the International Criminal Tribunal for the former Yugoslavia (ICTY) and the International Criminal Tribunal for Rwanda – has added to this body of law by clarifying the classification of conflicts and writing out in greater detail the kinds of protections civilians should receive (Deeks, 2014). Yet despite this extensive framework, wartime adjudication often goes beyond what IHL provides. Domestic courts usually remain operational especially during prolonged conflicts, continuing to handle cases that form part of everyday life – registering births, dissolving marriages, or resolving social benefit or property disputes. The persistence of courts during conflict – hearing both extraordinary cases and the mundane disputes of daily life – suggests that legality is not silenced but reconfigured.

The logic of subordinating civil and human rights to security imperatives during emergencies, which could be heard in Cicero's maxim and has been a long-standing tradition of U.S. ‘crisis jurisprudence’ (Garrison, 2011), has a formal counterpart in the European context (Gross and Aoláin, 2006) through Article 15 of the European Convention on Human Rights (ECHR), which permits derogations in times of ‘war or other public emergency threatening the life of the nation’. Ukraine first invoked this provision in 2015, following the annexation of Crimea and the outbreak of armed conflict in Donbas. In March 2022, following Russia's full-scale invasion, it extended derogations nationwide and expanded them to 11 Convention provisions, including freedoms of expression, assembly, and association, as well as several Protocol rights (Apostol, 2022), and several derogations from the International Covenant on Civil and Political Rights (ICCPR).^1^ Importantly, in April 2024 Ukraine narrowed the scope of its derogation (both from ECHR and ICCPR), restoring guarantees such as freedom of religion, non-discrimination, and the right to an effective remedy (Council of Europe, 2024). This recalibration demonstrates that the onset of military conflict is not uniformly detrimental to courts. Rather, it may serve as a critical juncture (Epperly and Sievert, 2019) – an inflection point that can yield either negative or positive trajectories (including gradual re-extension of rights protections). This raises a broader empirical and theoretical puzzle: how do Ukrainian judges themselves adjudicate under conditions of war? Building on comparative debates about judicial behaviour in conflict, this paper examines the Ukrainian case to illuminate how judges navigate this tension in practice.

Our central argument is that war, rather than silencing Ukraine's judiciary, has activated a distinct form of judicial agency: a strategic turn to international legal frameworks when domestic legislation, shaped by national security imperatives, limits judges’ ability to protect civil and human rights. Drawing on Kubal's (2024) insights into judicial responses to authoritarian backsliding, we show how Ukrainian judges, under existential threat, similarly step beyond legal formalism to invoke international legal instruments – particularly the Namibia exception (Higgins, 1972) and European Court of Human Rights (ECtHR) case law – to adjudicate everyday though often complex wartime claims, especially from occupied territories. While off-bench, Ukrainian judges remain embedded in the everyday realities of war – facing the same physical dangers, displacements, and emotional burdens as their fellow citizens – our on-bench analysis reveals how they have begun to innovate within their legal reasoning.

This paper makes both an empirical and theoretical socio-legal contribution. While focused on a single country, it relies on unprecedented empirical access to the lived experiences of Ukrainian judges during wartime. Theoretically, we conceptualise Ukrainian judges as legal bricoleurs – creatively drawing on international human rights norms to balance civil liberties, national security, and bureaucratic hurdles. Furthermore, and unlike other Eastern European contexts, where judicial independence has been undermined in peacetime (Bojarski, 2025; Kovács and Scheppele, 2018; Puleo and Coman, 2024), the Ukrainian case adds new depth to our understanding of judicial behaviour under extreme pressure and expands current debates on legal resistance, judicial agency, and the limits of legal formalism and bureaucratisation (Kurkchiyan, 2013) in times of crisis. The paper is organised into three main parts. Part One offers a two-pronged literature review: first, situating Ukraine's judiciary within the post-2014 regional context and engaging critically with scholarship on legal formalism, legal dualism, and the protracted trajectory of judicial reform; and second, placing the Ukrainian judiciary in comparative perspective alongside courts in other jurisdictions (United States, Israel–Palestine, Latin America, Northern Ireland, and Spain) that have operated under conditions of war and armed conflict. Following a discussion of methodology, Part Two presents the empirical findings, examining both the off-bench experiences and on-bench decision-making of judges during the Russian invasion. Part Three analyses how judges respond to wartime disruption by acting as *legal bricoleurs* – drawing on international human rights norms to navigate legal uncertainty and institutional fragility. This analysis shows how such practices complicate prevailing assumptions about the limits of judicial agency in times of crisis, revealing war not only as a site of disruption but also as a generative context for legal innovation with long-term implications for Ukraine's transitional justice and its place within the international legal order. The paper concludes by linking these findings back to broader theoretical debates.

## The Ukrainian Judiciary Through Post-Soviet Legacies

Understanding the contemporary Ukrainian judiciary requires situating it within the broader context of post-Soviet legal transformation with another round of substantial judicial reform, which started in 2016, shortly after Revolution of Dignity in 2014. Two persistent legacies have shaped judiciary's institutional character and day-to-day functioning: legal dualism (Hendley, 2011; Kyselova, 2014; Ledeneva, 2008) and bureaucratic legal formalism (Cserne, 2020; Galligan and Matczak, 2007; Kurkchiyan, 2013; Mańko, 2013; McBarnet and Whelan, 1991). These legacies, deeply rooted in Ukraine's Soviet past and exacerbated by the fragmented nature of its political elite, continued to undermine judicial independence and obstruct meaningful legal reform.

### Legal Dualism and Political Interference

Judicial independence – widely regarded as a cornerstone of the rule of law – has long remained Ukraine's central post-Soviet challenge (Kyselova, 2014: 178). Despite pressure from the EU and Council of Europe, reforms have consistently fallen short (Rechberger, 2009). The ECtHR's early judgment in *Sovtransavto Holding v. Ukraine* (2002, application no. 48553/99) identified systemic failures – executive interference, inconsistent legal application, and appeal procedures undermining finality (Wilkinson, 2003) – that persist more than two decades later.

The concept of legal dualism helps explain these deficiencies. Hendley (2011) describes post-Soviet courts as operating on two tracks: one visible and rule-bound, the other informal and politically manipulated. Kyselova (2014) shows how in Ukraine,

neither the identity of the parties, nor the subject-matter of the dispute can unambiguously determine which track any specific case is likely to follow … This makes an *ex ante* distinction between political and mundane cases nearly impossible (Kyselova, 2014: 198–199).

She argues that dysfunctions such as delays and corruption are not transitional weaknesses but a *deliberate distortion* serving elite interests. Darden (2008) similarly notes that low salaries and a logic of graft, where corruption is not merely tolerated but strategically deployed by political actors as a tool of control – make judges dependent on political patrons (Darden, 2008: 35).

This dualism has been further entrenched by Ukraine's fragmented political environment. Unlike Russia, where centralised authority has enabled the ruling elite to maintain vertical control over the judiciary, Ukraine's political landscape is characterised by intense competition and shifting alliances (Kuzio, 2005; Oliinyk and Kuzio, 2021). Political fragmentation has resulted in what Kyselova described as the proliferation of ‘pocket judges’ – jurists loyal to particular business or political figures (Kyselova, 2014: 187; see also Popova, 2010; Trochev and Ellett, 2014). Under wartime conditions, however, pressures increasingly stem from security institutions, with civil liberties at risk of subordination to the priorities of the war effort.

### Legal Formalism as a Legacy of Bureaucratisation of Justice

In tandem with legal dualism, the Ukrainian judiciary also exhibits a strong tradition of legal formalism and bureaucratisation – a mode of reasoning that privileges rigid adherence to the letter of the law over purposive or value-based interpretation (Kurkchiyan, 2013). This tendency is not unique to Ukraine; it is shared across much of post-communist Central and Eastern Europe (CEE) (Cserne, 2020; Mańko, 2013). As Kubal (2024) notes, many CEE judiciaries inherited a legal culture in which law was subordinate to political authority. With the collapse of state socialism and the introduction of judicial independence after 1989, judges across the region retreated to the safety of legal texts, interpreting them narrowly to avoid any perception of political bias or activist overreach.

This cautious posture has deep roots. Denis Galligan and Marcin Matczak's study of Polish courts during the 1990s famously described judges as ‘the mouthpieces of the law’, reflecting their commitment to black-letter legalism (Galligan and Matczak, 2007). A similar logic permeates the Ukrainian judiciary, where legal formalism is reinforced through a deeply embedded bureaucratic culture. As Marina Kurkchiyan observes, Ukrainian civil procedure – modelled on the Soviet variant of the continental tradition (Maleshin, 2007: 543) – places heavy reliance on written documentation, often making judicial outcomes contingent not on judges’ interpretive agency but on litigants’ capacity to navigate adjacent bureaucracies such as welfare offices, health services, or building inspectorates (Kurkchiyan, 2013: 519). In this environment, courts functioned less as autonomous adjudicators and more as integral cogs in a wider state machinery, with bureaucracy becoming not just a method of operation, but the very social identity of the judiciary (Kurkchiyan, 2013: 525). Formalism, in this context, is not merely a procedural habit but a professional ethos that defines what judges believe their job to be.

Yet, as Kubal argues, this reliance on formalism and bureaucratisation also limits the judiciary's capacity to respond to new and complex social realities, especially under conditions of crisis (Kubal, 2024). In Ukraine, where bureaucratisation and formalism exists alongside pervasive leal dualism, judges often navigate a paradoxical environment – applying the law rigorously in some cases, while in others adapting their decisions in response to implicit political or economic pressures. This tension not only constrained but became inalienable feature of judicial agency, ultimately undermining both the perceived legitimacy and the substantive independence of their rulings.

### Judicial Reach to International law After 2014: The Post-Maidan Uptick

The aftermath of the 2014 Revolution of Dignity brought renewed expectations for Ukraine's judiciary to overcome entrenched legacies of legal dualism, formalism, and corruption (Lashyn et al., 2023: 1489–1490; Popova and Beers, 2020: 113–114). Early legislative measures, such as the 2014 Law ‘On Restoring Trust in the Judiciary’ – aimed at removing judges complicit in the repression of protestors – offered symbolic change but failed to address deeper systemic vulnerabilities (Wilson, 2017). Judicial decision-making remained susceptible to informal political and economic pressures, reflecting the persistence of dual-track operations in which formal legal norms coexisted with extra-legal influences (Halushka and Shevchuk, 2021).

Under increasing pressure from international donors and EU partners, Ukraine initiated a more comprehensive judicial overhaul in 2016. This included the restructuring of the court system, the introduction of automatic case distribution to reduce corruption risks, and the creation of two key governance bodies: the High Council of Justice, tasked with judicial appointments and disciplinary oversight, and the High Qualification Commission of Judges (HQCJ), responsible for recruitment and vetting (Chyzhyk, 2022). However, both bodies quickly encountered internal resistance, when confronted with persistent legal dualism. The HQCJ became inactive in 2019, stalling judicial appointments and exacerbating case backlogs (Chyzhyk, 2022).^2^ Maria Popova and Daniel J. Beers went as far as to proclaim ‘no Revolution of Dignity for Ukrainian judges’ due to weak commitment of political elite to judicial reform and the absence of an influential reformist group within the judiciary (Popova and Beers, 2020: 130).

One key area of reform advocacy, which brought tentative results has been the judicial engagement with international law, particularly human rights jurisprudence from the ECtHR and the Court of Justice of the European Union (CJEU), especially after signing the EU-Ukraine Association Agreement (OJ L161) as the basic legal framework of EU– Ukraine relations on 27 June 2014. Yet, as several scholars point out, structural and doctrinal limitations continued to constrain this nascent engagement.

As authoritatively explained by Roman Petrov within the Ukrainian legal system ratified international treaties rank below the Constitution but above secondary legislation (Petrov, 2020, 2018). This means that while such treaties can override statutory law, they cannot invalidate conflicting constitutional norms. This hierarchy has been codified in Ukrainian legislation and consistently reaffirmed by the Constitutional Court (Petrov, 2018: 101). Thus, even when judges are motivated to apply international law, their hands may be tied when domestic constitutional provisions diverge from international standards.

Beyond formal hierarchy, there are interpretive and cultural barriers to international law's judicial incorporation. As Wilkinson (2003) observed in their early empirical study of Ukrainian legal practice, domestic judges and jurists often neglected international human rights jurisprudence due to (1) a widespread but erroneous belief that case law was irrelevant in civil law traditions, (2) linguistic ambiguities in translating ‘case law’ into Ukrainian legal discourse, and (3) the scarcity of accessible translations of international judgments (Wilkinson, 2003: 223). These barriers reinforced a long-standing professional orientation towards textual formalism, leaving little space for doctrinal creativity or rights-based reasoning (Ivanets, 2024).

A clear illustration of the tension between international obligations and domestic constitutional constraints is *Polyakh and Others v. Ukraine* before ECtHR (2019, application nos. 58812/15, 53217/16, 59099/16, 23231/18, 47749/18). After the 2014 Euromaidan, Ukraine's lustration law barred individuals linked to the Yanukovych regime from public service. Officials – including judges and prosecutors – could be dismissed without individualised review. Domestic courts declined to apply Convention standards directly, deferring instead to constitutional procedures, which insulated the law from international scrutiny for years. The ECtHR later found that Ukrainian courts could have engaged with Convention jurisprudence on blanket exclusions, revealing not incapacity but a preference for procedural deference over substantive rights analysis. As Mykola Gnatovskyy (Ukrainian judge at the ECtHR since 2022) noted:

This situation is remarkable as the Strasbourg Court has effectively done the job of Ukraine's Constitutional Court, which had not spelled out its views, despite having been requested to do so several times by the Supreme Court of Ukraine and by members of Parliament back in 2014 and 2015 (Gnatovskyy, 2022: 447–448).

A similar stance appeared in *Selygenenko and Others v. Ukraine* (2021, application nos. 24919/16 and 28658/16), where internally displaced persons from Crimea and Donetsk and Luhansk oblasts were excluded from local elections due to strict residence rules. Domestic courts upheld the exclusion by literal reliance on electoral legislation, ignoring applicants’ ties to their host communities. The ECtHR ruled this discriminatory under Article 1 of Protocol No. 12, faulting courts for neglecting equality principles and failing to harmonise domestic law with the Convention. While reforms followed, it was Strasbourg that secured rights protection. Together, these cases highlight a persistent formalism in Ukrainian adjudication: judges acknowledge international norms rhetorically but retreat to domestic orthodoxy when law and rights collide, leaving enforcement to the ECtHR.

Despite these constraints, the post-2014 period did witness some openings. Petrov's empirical research into judicial references to the ECtHR and CJEU – drawing on Ukraine's Unified State Register of Judicial Decisions – showed a marked increase in citations between 2006 and 2019, with more than 130,000 mentions recorded, primarily by administrative courts (Petrov, 2020: 194). These references often occurred in the context of ‘Chornobyl social protection’ disputes^3^ or cases affirming that citizens may rely on the state's obligations, even in cases when these obligations are provided in law without direct effect (referring to the case law on the principle of direct effect of EU directives invoking *Van Duyn v. Home Office.*^4^) However, as Petrov noted, the EU-Ukraine Association Agreement did not confer on Ukrainian judges the formal requirement to ‘possess general knowledge on the foundations of EU law and CJEU case law’ (Petrov, 2020: 189) and most of these references amounted to formulaic copy-paste citations, with little evidence of substantive legal reasoning or adaptation. In some cases, Ukrainian judges even misattributed CJEU cases to the ECtHR, further underscoring the shallow and formalistic nature of this early engagement (Petrov, 2020: 190).

Nevertheless, moments of more normative alignment with European values did surface. A striking example can be found in the judgment No. 346/3499/16-c of the Interdistrict Court of Kolomyia (7 July 2016), which proclaimed:

After the signing of the Association Agreement with the European Union by the President of our country, and after ratification by the Verkhovna Rada [Parliament] of Ukraine, Ukraine, as a state aspiring to full membership in the EU, must respect the private property rights of every person as a basic tenet and a cornerstone of European values and inviolable foundation of the EU, which must be complied with by all Member States and by associated countries (cited after Petrov, 2020: 192).

Such normative statements reflect a nascent form of judicial agency in the spirit captured by Kubal's (2024) work with Polish judges – an affective and discursive commitment to European integration – that diverges from the inherited logics of formalism, bureaucratisation and political dependency.

## Judging During war

The escalation into full-scale war in 2022 after Russia's military invasion presents an even more complex terrain for understanding judicial behaviour. As Epperly and Sievert (2019) observe, conflict onset operates as a *critical juncture* – a moment of rupture that unsettles institutional equilibria and recalibrates the distribution of power between branches of government (701). In these contexts, courts are often expected to defer to the executive, prioritising state security over rights. This assumption is visible in the US tradition of crisis jurisprudence, where judges repeatedly endorsed extraordinary powers during wartime (Garrison, 2011; Gross and Aoláin, 2006). In *Hirabayashi v. United States* (1943), Chief Justice Stone justified the curfew imposed on Japanese Americans by invoking military necessity, while Justice Douglas insisted that ‘peacetime procedures do not necessarily fit wartime needs’. The decision illustrates how judges themselves articulated a logic of exceptionalism, embodying what critics later described as abdication of the judicial role in times of emergency (Dudziak, 2010; Irons, 2012, Neuborne, 2005).

Yet comparative scholarship demonstrates that this deferential model is far from universal. Courts in different conflict settings have often acted as sites of negotiation between security imperatives, political pressures, and rights protections. In Israel, for example, Amnon Reichman (2011) describes a form of ‘bounded independence’, whereby the Supreme Court exercised judicial review over military decisions while remaining constrained by domestic politics and international scrutiny. In this way, judicial independence becomes conditional, flexible, and constantly negotiated – a far cry from the binary assumptions of either full autonomy or total subservience. Gad Barzilai (2007) further highlights the silence of otherwise activist legal communities on Palestinian occupation-related matters, showing how judicial engagement is both selective and strategic. The result is a judiciary that performs legal legitimacy, sometimes innovating in its reasoning, while also accommodating the structural imperatives of a security state.

Other comparative cases, particularly relating to transitional justice post-armed conflict, further highlight judges’ positioning between deference and innovation. In engaging this comparative body of work, we have deliberately expanded the notion of ‘war’ to include non-international conflicts, in order to situate Ukraine in a broader comparative frame. At the same time, we emphasise that the conflict in Ukraine is widely recognised as an international armed conflict, rooted in Russia's invasion of a sovereign state (Hauter, 2023; *Ukraine and the Netherlands v. Russia* [2025, Grand Chamber ECtHR, application nos. 43800/14, 8019/16, 28525/20 et al.]). Our use of literature on non-international conflicts is therefore intended not to reframe Ukraine's war nor delegitimise it, but to enrich analysis where direct analysis is scarce (Grant, 2014, Olson, 2014, Revkin et al., 2023).

Scholars of Latin America, for instance, trace how judges in Colombia engaged deeply in transitional justice processes: striking down amnesties and insisting that reduced sentences for paramilitaries were conditional on truth-telling (Drange, 2022). The Columbian Constitutional Court (CCC) reviewed peace frameworks, assessed amnesties, and advanced victims’ rights, effectively judicialising the peace process (Cepeda-Espinosa, 2004; Landau, 2021). Manuel Cepeda-Espinosa (2004) describes CCC judges pushing back against political and military pressures, while Bård Drange (2022) characterises this as a ‘tug of war’ between civil society and state-paramilitary collusion, in which the judges often sided with victims. Huneeus et al. (2010) argue that judges in the region acted as ‘strategic actors’, using legal opportunities to extend their authority and embed human rights discourse into political life. In Northern Ireland (NI), Jackson and Doran (1995) show how the removal of juries in Diplock courts transformed the judge into a central fact-finder, placing them in a more inquisitorial role. In a similar context, McEvoy and Schwartz (2015) examined how NI judges, under pressure from parliament, the public, and their peers during and after the conflict, adapted their decision-making and professional identities. They argued that judges were placed in a performative role, navigating expectations from multiple ‘imagined communities’ while facing personal security threats due to the sensitivity of their cases (McEvoy and Schwartz, 2015: 548). The authors highlight the importance of judicial self-reflexivity and suggest that moving beyond strict legal formalism in politically and historically charged contexts can better support transitional justice processes (McEvoy and Schwartz, 2015: 555). A different picture emerged, however, in Spain, where the enduring legacy of fascist-era legal formalism remained visible in the judiciary's approach to both historical and contemporary conflicts. As documented by Joxerramon Bengoetxea, the Spanish Supreme Court's reliance on Article 2 of the 1977 Amnesty Law highlights how formalist interpretations of legality continue to shield the Franco regime's crimes from judicial scrutiny: the Court maintained that ‘the right to know historical truth is no business of criminal procedure’ thereby denying the applicability of international human rights and criminal law norms (Bengoetxea, 2020: 599). The same formalist logic reappears in the treatment of the Basque conflict. As Philippe Duhart observed, Spanish courts broadened the legal definition of ‘terrorist organisation’ to encompass non-violent Basque nationalist groups, constraining their participation in peace-making efforts and reaffirming a punitive, state-centric criminal justice model (Duhart, 2020: 432–433). This posture reflects a positivist and procedural conception of law privileging institutional continuity and legal closure over substantive justice in conflict adjudication.

Bringing this back to Ukraine, the comparative literature underscores variation in how judges navigate conflict, but it offers few clear answers for the present war. Much of the existing scholarship – from U.S. crisis jurisprudence to Israeli ‘bounded independence’, to Latin American, Northern Ireland's and Spanish transitional justice experiences – demonstrates that judges under pressure rarely retreat uniformly; instead, they adapt, improvise, or strategically perform legitimacy. In the Ukrainian case, this raises unresolved questions: have judges largely deferred to the executive in the name of national security, or have they, like their counterparts elsewhere, carved out spaces of autonomy by creatively reinterpreting law? And how have post-Soviet legacies of legal dualism, formalism, and bureaucratisation shaped the scope of their agency? Our study suggests that Russia's invasion has forced Ukrainian judges into precisely this terrain of improvisation, where legal bricolage – drawing on international and European human rights law and jurisprudence – becomes a means of sustaining judicial agency under fire. The analysis that follows moves from theoretical debates to on-the-ground insights – tracing both the *off-bench* disruptions that shape judges’ professional lives and the *on-bench* strategies through which they reinterpret and reassert the rule of law.

## A Note on Methods

This study adopts an interpretive qualitative methodology, grounded in social constructivist and critical realist approaches (Archer, 1996; Berger and Luckmann, 1991) and is premised on the understanding that legal ideas and practices – particularly those relating to human rights and judicial independence – are shaped by actors operating within specific socio-historical and institutional contexts (Stammers, 1999).

The research design was informed by the first author's extensive experience conducting mixed-method, multilingual studies of law in everyday life in Eastern Europe and Russia. Given the transnational character of the project – engaging with both resident and displaced judges from Ukraine – the primary data collection was carried out fully by the second author, whose professional background in Ukraine and fluency in Ukrainian, Russian, and English enabled deep contextual engagement and rapport-building with participants.

Our research relied on snowball sampling, whereby initial interlocutors introduced us to colleagues and suggested further professional avenues. Access to the judicial community was particularly sensitive under wartime conditions, when judges faced overwhelming workloads, heightened security concerns, and public scrutiny. Some interlocutors were known to us from earlier professional collaborations, which facilitated contact; others we reached through ‘cold’ approaches identified via open sources. Unsurprisingly, the most reliable access occurred when we could refer to another judge's recommendation. Judges are often considered a ‘hard to reach’ group, and this was further amplified by the war: several who initially agreed later declined interviews, citing time pressures, security concerns, or unease after reviewing our research materials. These withdrawals are not merely obstacles but data in themselves, revealing how the war sharpens the conditions of (un)openness of the Ukrainian judiciary to external inquiry.

Privacy was a recurrent concern. Even among those who did participate, many insisted on confidentiality, highlighting the sensitivity of discussing judicial practice under conditions of conflict. The judges did not impose any conditions before the conversations (on what they should or should not be questioned), although we made clear that they had a right to decline to answer any questions they found uncomfortable. In practice, none of the judges chose to skip a question. However, in several instances they requested that certain information – typically details concerning particular cases or some nuances of judicial work – remain off the record. We respected this by excluding such material from the transcripts and by not subjecting it to analytical reflection. Our eventual sample was weighted toward human rights–conscious judges, some of whom had received ECtHR training or engaged as lecturers in such programmes (e.g., at the Council of Europe). While they did not self-identify as ‘activists’, their accounts often reflected efforts to align judicial practice with international standards. Several also undertook public initiatives, such as outreach to schools or peer training. It should be noted that these reform-minded, human-rights-oriented judges – representing the avant-garde of the Ukrainian judiciary – often tailor their messages to multiple audiences: primarily to their fellow judges, whom they seek to persuade from within, but also to the broader public, aiming to make the judicial system more transparent and accessible to ordinary citizens. This introduces a sampling bias, privileging reform-minded voices, but also illustrates how wartime pressures intersect with ongoing attempts to reshape the judiciary from within.

Empirically, the research draws on 17 in-depth, semi-structured interviews with Ukrainian first instance court judges conducted between January and August 2024.^5^ Each interview lasted between 60 and 180 min and was conducted in Ukrainian. The interview sample had a 1:3 male-to-female ratio, which we consider representative given that women make up 47.9% of first-instance court judges (Fuley, 2016: 44) and due to ongoing war. The sample was purposively selected to include judges from various jurisdictions across Ukraine, with an emphasis on those who had experience working in courts located within 60 kilometres of the pre-2022 conflict line in the Donbas region. One illustrative case includes a judge from *Z*, a town situated 30 kilometres from the demarcation line, who played a pivotal role in issuing Ukrainian identity documents to civilian population from the occupied territories, and which court was later subject to total destruction by Russian forces in October 2024. For the safety of our interviewees, all names were anonymised to protect their identities. We preserved only the type of court, gender of the judge, and the broad geographical location of the court (West, East, Centre, North, South, with Kyiv indicated separately given its unique administrative status).

The positionality of the interviewing researcher and co-author – a native Ukrainian speaker – did shape these encounters. This helped to capture more directly language and cultural nuances – often intensified by wartime discourse – and made it easier to catch some references and nuances hinted ‘in-between lines’ by judges, which normally might have been lost in translation. Although our initial interview guide was focused on engagement with ECtHR practice in ordinary judicial work, the wartime context permeated the conversations. Even when not directly asked, judges linked international human rights law to emergency situations in which domestic legislation lagged behind unfolding realities.

Interviews were transcribed verbatim and analysed thematically. Thematic coding was guided by an interpretive framework attentive to both personal and professional dimensions of judicial life under wartime conditions (Braun and Clarke, 2006). Our interpretative methodology foregrounds judges’ own framings of their practices and constraints. We consider this innovative, as it moves beyond institutional reform or jurisprudential, textual analysis to illuminate how judges themselves describe incorporating international law into daily practice under the extraordinary disruptions of war. These included physical threats, displacement, institutional collapse, new responsibilities for the population from occupied territories, and the burden of increased caseloads due to the suspension or relocation of courts from occupied areas.

These methodological reflections highlight both the promise and the limits of qualitative research with judicial elites in conflict settings: access is partial, voices are selective, and findings reflect not only the fragility of institutions but also the resilience and bricolage of judges working *inter arma*.

## Off-Bench Realities at the Onset of Russia's Full-Scale Invasion

Mainstream scholarship often treats war as a state of exception, a suspension of legality in which sovereign power overrides the juridical order (Agamben, 2004). But as Mary Dudziak (2010) has critically pointed out, treating war as an interruption to legal normalcy blinds us to the continuity of war in the lived experience of legal actors, whereby ‘viewing war as an exception to normal life leads us to ignore the longstanding persistence of war’ (Dudziak, 2010: 1670). For the Ukrainian judges we interviewed – many of whom have operated under conditions of occupation, displacement, and disruption since the 2014 annexation of Crimea and the war in Donbas – this observation rang particularly true. Civilian courts have continued to serve as the backbone of Ukraine's justice system even under martial law, sustaining high clearance rates despite bombardment and budget cuts (European Commission, 2024; Kent, 2023). Yet behind this institutional resilience lie profound disruptions to judges’ everyday lives.

Displacement was a recurring theme in our interviews, echoing Elena Fiddian-Qasmiyeh's (2016) observation that displacement reverberates long after relocation through insecurity, reduced quality of life and a profound sense of social dislocation. One judge described the absence of state guidance in the early days of invasion:

When the war began, we weren't evacuated, they didn't tell us anything, we made decisions ourselves on what to do (…) I was in the court's common room and told colleagues: ‘War has started, what do we need to do? My children are near the office. We need to get the children out’ (first instance court, female judge, Kyiv).

Others echoed this existential fragility. Despite the severity of the threat, judges were not informed or prepared in advance of the full-scale invasion. They were formally instructed about what to do in case of occupation almost three weeks after the beginning of the full-scale invasion – on 13 March 2022.^6^ One judge from Kyiv region bitterly remarked on her experience of being abandoned by her government:

We see releases [of judges] from captivity. But the question is, how did this judge get captured? A judge who considered cases on ‘DNR people,’ how could it be allowed for her and her family to be captured? (first instance court, female judge, Centre).

The risks to personal security remain acute. Judges spoke of colleagues killed, courts destroyed, and staff targeted by drones – as of April 2023, 12 judiciary system representatives were killed as a result of the Russian attacks (Adamska-Gallant et al. 2023: 17). Courts near the frontlines face direct targeting, and personnel have suffered attempted assassinations. These accounts resonate with feminist critiques of the artificial divide between public and private, revealing how caregiving (evacuating the children), survival, and judicial labour collapse into a shared field of responsibility (Fineman, 2005; MacKinnon, 1989).

Workload was another heavy burden. As entire jurisdictions were relocated or incapacitated (Adamska-Gallant et al. 2023; Chyzhyk, 2022; Kent, 2023), judges in safer areas inherited vast backlogs of cases:

Since the beginning of the full-scale invasion, we were assigned jurisdiction over three courts … And we were literally drowning in cases (first instance court, female judge, East).

As Cardinal (2025) shows in her work on the Free Syrian Judicial Council, continuing to adjudicate amid bombardment can itself be a form of judicial resistance. Ukrainian judges share this ethos of persistence, but with one crucial difference: they resist from within state institutions rather than outside them. In doing so, they transform ordinary judicial acts – convening hearings, writing judgments, mentoring staff – into quiet assertions of legality and continuity under fire:

[Upon evacuating my children] I returned, because I had to announce a verdict that day (first instance court, female judge, Kyiv).

Those for whom the war has taken now a decade of professional life, seem to agree with Dudziak that ‘law during war can be seen as the form of law we in fact practice, rather than a suspension of an idealised understanding of law’ (Dudziak, 2010: 1670). Even amid exhaustion, judges described rituals of continuity:

And every day, when we went home, we took our robes, badges, books we lived by… (first instance court, female judge, Kyiv).

These routines, however mundane, become acts of defiance. Judges’ ability to maintain professional integrity while managing fear, family responsibilities, and logistical uncertainty speaks to a kind of resilience embedded in the everyday (De Certeau 1984). Ukrainian judges’ stories therefore do not fit neatly into Agamben's paradigm of exception or the U.S. model of crisis jurisprudence. Rather, they reveal continuity amid collapse, resilience amid insecurity, and the embodied endurance of judges – often women – who sustain legality while keeping their families safe.

## On-Bench Judicial Agency: Turn Towards International law

Amid the complexities of war and occupation, Ukrainian judges have stepped into a remarkable and often understated role: applying international legal norms – sometimes in defiance or absence of national legislation – to safeguard the civil and human rights of citizens from occupied territories. In doing so, they have transformed the courtroom into one of the last remaining spaces of legal legibility for populations caught in geopolitical limbo. Their actions speak not only to the creative use of legal reasoning but also to a deeply rooted professional and ethical commitment to justice.

Nowhere is this more visible than in the work of judges who developed procedures to recognise births, deaths, and other vital events for Ukrainians residing in the so-called Donetsk People's Republic (DNR) and Luhansk People's Republic (LNR).^7^ These were not exceptional cases; they were daily occurrences. ‘We had a flow, every day we had ten such cases’, judge XY, presiding a court in the front-line town of Z., explained. People were arriving daily from occupied territories with medical certificates and documents issued by local institutions that Ukraine did not officially recognise. Yet these papers were often the only proof they had that a child had been born, or a parent had died. Without Ukrainian birth or death certificates, individuals could not access healthcare, social services, or even move freely.

One of the key legal instruments regulating the status of individuals in occupied Ukrainian territories was Law No. 1207-VII, ‘On Ensuring the Rights and Freedoms of Citizens and the Legal Regime in the Temporarily Occupied Territory of Ukraine’, adopted on 15 April 2014. Article 9 of this law invalidated any documents or decisions issued by occupation authorities, meaning such acts had no legal consequences within Ukraine's legal order. To address the resulting legal limbo, subsequent legislation – the 2016 Law No. 990-VIII (On Amendments to the Civil Procedural Code of Ukraine Regarding the Establishment of the Fact of Birth or Death of a Person in the Temporarily Occupied Territory of Ukraine) introduced a judicial mechanism for establishing the fact of birth or death in occupied territories, requiring individuals to first obtain a court decision before approaching the Registry Office. While a 2018 amendment allowed limited recognition of such documents (Law No. 2268-VIII), the procedure remained unchanged in practice. Applicants often had to solicit a written refusal from the Registry Office before submitting their court application. Although a 2021 Supreme Court explanatory letter (No. 985/0/208-21) clarified this was unnecessary, and 2022 amendments to Law No. 1207-VII sought to streamline the process, at the point of writing this paper, courts remained essential intermediaries in acquiring basic identification documents.^8^

This labyrinthine system of births and deaths registrations for Ukrainian citizens on the occupied territories reflected what Kurkchiyan (2013) dubbed as a ‘justice through bureaucracy’ model – an extreme form of legal formalism in which procedural correctness overrides accessible or equitable outcomes, where even the most justice-minded judges are obstructed by the opaque and unyielding bureaucracies embedded across other branches of the state (Kurkchiyan, 2013: 522). Faced with a domestic legal framework that failed to account for the lived realities of people in occupied territories, some judges, like XY, looked to international human rights law for normative guidance – quietly reasserting judicial agency through flexible reasoning and rights-based interpretations that allowed them to uphold justice where national law fell short:

We referred to the Namibia exceptions … certificates issued by medical institutions should be [valid as] evidence in judicial institutions (first instance court, female judge, East).

The foundational concept invoked was the Namibia exception, a principle originally articulated in the context of South West Africa under apartheid rule. The exception allowed courts to consider documents issued by de facto authorities – not to recognise the legitimacy of those regimes, but to avoid harming individuals by denying them access to civil status and legal identity (Higgins, 1972). The legal roots of this principle trace back to the 1971 Advisory Opinion of the International Court of Justice (ICJ) concerning the continued presence of South Africa in Namibia after the termination of its mandate. Following United Nations resolutions condemning South Africa's administration of the territory, the ICJ found this occupation illegal and instructed all States not to recognise acts performed by South African authorities on behalf of Namibia. However, it also clarified that this non-recognition must not impede the rights of individuals. As Rosalyn Higgins (1972) emphasises, the Court drew a crucial distinction between political legitimacy and the human consequences of legal voids, thereby allowing for limited functional dealings with de facto regimes to protect civilians’ rights.^9^ The Namibia exception, which emerged from a decolonial struggle, was adapted by Ukrainian judges to navigate the legal limbo created by Russia's occupation, demonstrating the enduring applicability of international legal reasoning born out of anti-colonial contexts to contemporary wartime governance:

People who came, went through these 10 circles of horror – they come and nothing … For a child – this means not being able to leave, no medical, social guarantees, no rights … (first instance court, female judge, East).

A bottom-up legal infrastructure emerged in courts near the frontlines to address the urgent needs of individuals seeking Ukrainian legal recognition of births and deaths. People would often arrive for just a single day, carrying documents and needing swift resolution. Court staff, sensitised to the immediacy of these cases, would assess whether the individual had accommodation or needed to return the same evening. If the latter, the case was prioritised and scheduled immediately, sometimes within hours:

We created our own well-established practice … we tried to do everything quickly. The girls [in the court] were already trained, they asked visitors if they had a place to stay … if not – we tried to do everything in one day (first instance court, female judge, East).

Court assistants helped applicants on the spot, providing sample applications and guiding them through completion. They contacted local registry offices in advance to ensure documents could be issued the same day, and worked within tight deadlines –often extending past official hours:

We called them [the Registry Office] and said: ‘We have a person who has nowhere to stay, who needs to return back today, please wait, we're trying to do this quickly now’ (first instance court, female judge, East).

Judges would then conduct an expedited review, and rather than dismissing the DNR or LNR certificates due to their origin, they were assessed in conjunction with other materials such as Ukrainian passports of close family members, personal photographs, and oral testimonies.

This line of reasoning was also affirmed by judge AB in Kyiv region, who underscored the ECtHR's position that, despite Russia's effective control over the separatist territories –‘established beyond reasonable doubt’ (*Ukraine and the Netherlands v. Russia*, Grand Chamber, 2025) – courts must assess evidence in its totality, rather than rejecting documents solely on the basis of their origin. As judge AB noted, ‘the court uses as evidence certain documents that do not have legal force, but uses them alongside other documents’ (first instance court, male judge, Centre). This illustrates how the ECtHR's jurisprudence^10^ was not merely referenced but actively operationalised within judicial practice:

you cannot close your eyes to those documents (…) How then can a person protect their rights? (…) On the one hand, national legislation cannot a priori say that these documents are appropriate. On the other hand, the practice of the European Court [of Human Rights] says that such documents should be evaluated in conjunction with other documents, including statements of this person in a court session (first instance court, male judge, Centre).

This was more than just a workaround – it was a quiet legal rebellion against a system that, through inaction or hesitation, had made it easier for people to accept Russian documents than to retain Ukrainian ones:

We wrote our decisions that they are [Ukrainian] citizens, they should receive citizenship, they should receive a birth certificate… Why are we creating obstacles for us to have more citizens? (first instance court, female judge, East).

Through their close work with civilians from Russian-occupied territories – many of whom travelled across dangerous frontlines to seek Ukrainian legal recognition of life events – judges developed a sharpened sensitivity to the complex realities of survival under occupation. A judicial encounter with these individuals created a rupture in official rhetoric:

People have various reasons to stay in occupied territories. Some have sick or elderly parents there, some have health problems themselves, and some simply don't have the means to start a new life elsewhere. If the state could say: ‘You leave, and you will be guaranteed certain conditions,’ perhaps the situation would be different. And this doesn't even take into account the bullying and stigmatization that [internally] displaced persons face (first instance court, female judge, West).

Acting as legal bricoleurs, Ukrainian judges stitched together international norms, unrecognised documents, and personal testimonies to navigate legal voids – an improvisational practice that was essential not only for safeguarding individual rights in occupied and transitional spaces, but also activated a distinctive form of judicial agency: One characterised by pragmatic, rights-oriented decision-making that leverages international frameworks when domestic law falls short.

## Discussion: Judges as Legal Bricoleurs

The concept of *bricolage*, first introduced by Claude Lévi-Strauss in *The Savage Mind*, describes the process of assembling new systems of meaning from pre-existing, available materials – highlighting both the creativity and constraints inherent in working with what is ‘at hand’ (Lévi-Strauss, 1966 [1962]). Roger Bastide later expanded this concept, particularly in the context of colonial domination, arguing that bricolage serves not only as a creative response to scarcity but also as a means of resisting structural domination (Bastide, 1970). This understanding offers a compelling analytical lens for interpreting the Ukrainian judiciary's wartime legal practices. Faced with a debilitating legal vacuum – where rigid domestic legislation failed to account for civilian realities under Russian neo-colonial domination in Eastern Ukraine (Mälksoo, 2023) – judges mobilised international human rights norms and their own legal agency to protect individuals otherwise stranded in legal limbo. Much like Bastide's account of subaltern groups reconfiguring dominant cultural elements to reclaim agency, Ukrainian judges reassembled available legal norms to navigate the profound disjuncture between state imperatives and humanitarian needs. This legal bricolage thus reflects not only practical problem-solving but also normative resistance – an assertion of legal sovereignty and individual dignity amid systemic disruption.

The concept of legal bricolage has thus far been used to account for South Africa's post-apartheid legal trajectory, where a bricolage approach to justice deliberately blended criminal trials, truth commissions, conditional amnesties, and selective prosecutions (Beresford and Wand, 2020; Dent, 2023). As conceptualised by Alexander Beresford and Daniel Wand, the process of legal bricolage engaged in by transitional justice actors involved adapting both new and inherited legal tools, values, and practices to meet immediate challenges (2020: 534). In contesting the dominant prosecutorial model favoured by the International Criminal Court (ICC) together with its emphasis on criminal accountability, South African transitional justice actors adapted international norms to local political realities, demonstrating that global legal standards carry a core set of principles but can be operationalised through diverse institutional forms (Beresford and Wand, 2020: 554).

A different but complementary strand of work comes from the Democratic Republic of the Congo. Cirimwami and Magadju (2021) show how Congolese judges, facing inadequate domestic legislation, directly applied the Rome Statute in wartime rape prosecutions. This ‘legal translation’ illustrates how judges in fragile systems can improvise by importing international norms to fill gaps. Taken together, these examples reveal the two sides of legal bricolage: at times international norms are adapted to fit local political conditions, while at other times judges reach outward when local frameworks are insufficient. Yet Ukrainian bricolage differs in important respects. Whereas Congolese judges drew on instruments immediately available within the Rome Statute, Ukrainian judges have reached for norms less directly accessible – such as the Namibia exception – incorporating them into domestic adjudication as part of a broader struggle against neo-colonial domination. Here bricolage involves not only translation but also the selective activation of international legal resources to decolonise the practice of judging itself.

Work on international courts offers another vantage point. Shane Darcy's *Judges, Law and War* (2014) shows how judges at the ICTY, ICJ and other tribunals engaged in ‘law-creating practices’ when existing IHL failed to address new forms of conflict. Cases such as *Tadić* broadened the definition of armed conflict to encompass ‘protracted armed violence’ between states and organised groups (Darcy, 2014: 83), while human rights principles were blended into IHL to define concepts like torture and inhumane treatment: ‘the application of human rights law during armed conflict, including during situations of occupation, has been in many ways a judge-led endeavour’ (Darcy, 2014: 103). Though initially criticised as judicial activism, many of these interpretations are now embedded in the fabric of IHL. Darcy's jurisprudential analysis highlights the doctrinal and institutional impact of judicial creativity in shaping customary international law. Our study builds on this line of inquiry but extends it empirically: rather than focusing solely on appellate judgments and doctrinal reasoning, we examined how Ukrainian judges themselves understand and enact bricolage in daily practice.

Taken together, these comparisons suggest that Ukrainian judges are neither passive bureaucrats (Cserne, 2020; Kurkchiyan, 2010, 2013) nor simple enforcers of law. Like the South African transitional justice actors, international judges at the ICTY, or Congolese military tribunals, they adapt available materials under constraint. To be clear, much of this ‘innovation’ is not originating on the bench but percolates into courts through a wider ecosystem of human-rights mobilisation and training. Since the 1990s, organisations such as the Kharkiv Human Rights Group and the Ukrainian Helsinki Human Rights Union – alongside institutional programmes (Council of Europe and OSCE trainings or the EU's Pravo-Justice modules) – have normalised the direct use of international human rights law by legal professionals, including judges:

The situation, at least for me, changed after the full-scale invasion. My colleagues and I began actively studying international humanitarian law and the practice of the ECtHR. The Ukrainian Helsinki Human Rights Union launched powerful courses, in which I am a participant … This significantly changes the attitude toward ECtHR practice. We can read anything and find it anywhere for references. But these courses allow direct communication with judges of the ECtHR and staff members – this changes your attitude (first instance court, female judge, 2024).

Our interviewees repeatedly traced their turn to ECtHR jurisprudence to this civil-society-led pedagogy and curricular change, suggesting that judicial bricolage is embedded in, and catalysed by, broader shifts in legal culture rather than individual bench-level initiative.

## Conclusion

This study has shown that Ukrainian judges, far from acting as passive bureaucrats, have become *legal bricoleurs* – assembling fragments of domestic and international law to sustain legality and dignity under conditions of existential threat. Their work demonstrates that war is not a suspension of law but a crucible of legal creativity (Dudziak, 2010), where courts reassert authority precisely at the moment it seems most fragile. The evidence presented here highlights how Ukrainian judges engaged international law as a normative compass to bypass rigid or exclusionary provisions of secondary domestic legislation ill-suited to wartime realities. In cases such as registering births and deaths under occupation, judges improvised rapid, rights-based procedural innovations that challenged bureaucratic inertia and reasserted Ukrainian sovereignty in areas where it was most tenuous. These actions reveal a deeper judicial ethic: law's authority in wartime depends not only on institutional continuity, but also on judges’ willingness to adapt creatively in defence of individuals otherwise stranded in legal limbo.

Our argument contributes to broader comparative debates on judging in times of conflict. Like South Africa's transitional justice actors (Beresford and Wand, 2020), Ukrainian judges have blended inherited and novel tools to respond pragmatically to crises. Like the ICTY judges studied by Darcy (2014), they have engaged in practices that reshape legal categories and embed new standards of protection. And while Congolese tribunals demonstrated the translation of Rome Statute provisions into local adjudication (Cirimwami and Magadju, 2021), Ukrainian judges reached further – invoking norms not immediately at hand, such as the Namibia exception, to decolonise law's operation under Russian neo-colonial aggression.

The originality of this study lies in centring the lived perspectives of judges themselves, providing an empirical counterpoint to doctrinal accounts of wartime adjudication. It shows how everyday judicial acts – issuing verdicts, recording vital events, sustaining courtroom routines – become forms of legal resistance, transforming ordinary procedure into quiet assertions of sovereignty. By tracing these practices, we challenge dominant portrayals of post-Soviet judiciaries as inert and formalistic (Cserne, 2020; Kurkchiyan, 2010, 2013). Ukrainian judges, *inter arma,* demonstrate instead a pragmatic and rights-oriented judicial ethics, one that positions their courtrooms as crucial sites of innovation where legality is preserved not in spite of war, but because of it.

## Notes

1. Scholars, writing primarily from a normative perspective, have noted both strengths and weaknesses of this move: some considered these measures legally justified, as they met the Article 15 test of necessity, proportionality, and consistency with international obligations, while leaving non-derogable rights intact (Wallace 2020, Dzehtsiarou and Tzevelekos 2022). Others flagged procedural flaws and the ambiguous framing of the post-2014 conflict defined by Ukraine as an ‘anti-terrorist operation’, which potentially undermined legal defensibility (Denysov and Falalieieva, 2020; Milanovic 2015).

2. A legislative relaunch in 2021 aimed to restore these institutions with international oversight, but reform momentum was disrupted again by Russia's full-scale invasion in 2022. At the time, the HCJ was non-functional, and only resumed operations in 2023 (Adamska-Gallant et al. 2023).

3. The ‘Chornobyl social protection' disputes refer to a large body of administrative litigation in Ukraine stemming from laws that granted benefits – such as pensions, housing, and healthcare – to victims and liquidators of the 1986 Chornobyl disaster. Ukraine's systemic failure to enforce domestic court rulings in such cases led to a surge of lawsuits against government to the ECtHR and eventually the ECtHR's 2017 *Burmych and Others v. Ukraine* (Grand Chamber, application nos. 46852/13, 47786/13, 54125/13 et al.) decision, which struck over 12,000 applications calling for a general domestic remedy (*Yuriy Nikolayevich **Ivanov v Ukraine*, 2009, application no. 40450/04) rather than case-by-case adjudication. The ruling highlighted a deep judicial enforcement crisis and the challenges of legal integration when domestic compliance is lacking.

4. *Van Duyn v. Home Office* (Case 41/74, Judgment of 4 December 1974) is a landmark judgment by the CJEU concerning the free movement of workers between member states that affirmed the principle of direct effect of EU directives. The case involved a Dutch national denied entry into the UK due to her association with the Church of Scientology, which UK authorities considered socially harmful. The Court held that individuals could invoke provisions of EU directives before national courts even if the directives had not been fully transposed into domestic law, provided those provisions were sufficiently clear, precise, and unconditional.

5. According to judiciary reform in 2016, the four-tier court system (local courts, courts of appeal, high specialised courts, Supreme Court) in Ukraine was changed into a three-tier system: first instance courts (including district and city courts, local administrative courts, local commercial courts), courts of appeal, and the Supreme Court.

6. Recommendations for first instance and appellate courts in case of capture of a settlement and/or court or direct threat of its capture, Supreme Court, 13 March 2022, accessed from: https://ips.ligazakon.net/document/VSS00828?an=1 on 16 May 2025.

7. DNR and LNR are self-proclaimed Russian proxy regimes in eastern Ukraine. They declared autonomy from Ukraine in 2014 but are not internationally recognized as sovereign states.

8. As of 31 July 2023, the Ministry of Justice introduced a new digital mechanism allowing applications for birth certificates of children born under occupation to be submitted via email, with the option to receive a digital certificate and later obtain a hard copy upon presenting original documents (see: https://www.kmu.gov.ua/news/yak-zareiestruvaty-dytynu-iaka-narodylasia-na-tymchasovo-okupovanii-terytorii). While this marked a step toward easing access, court procedures remain necessary in cases lacking full documentation, and death certificates can still only be issued via court decisions.

9. For a historical account of litigating South African colonialism in Namibia before the ICJ, see Tzouvala (2020).

10. See the case of *Cyprus v. Turkey*, 2001, application no. 25781/94 and *Mozer v. The Republic of Moldova and Russia*, application no. 11138/10.
